# Supplementary material for: Chromothripsis during telomere crisis is independent of NHEJ, and consistent with a replicative origin
Source: Genome Res. 2019 May;29(5):737–49. doi: 10.1101/gr.240705.118 (PMC6499312; doi:10.1101/gr.240705.118)
Supplement: Supplemental Material [file supp_gr.240705.118_Supplemental_file_1.zip › contigs/annotated_contigs/DB89/contig.3.DB89_length_665_mean_cov_8.4045112782.docx]

**DB89_length_665_mean_cov_8.4045112782**

```
TTTTATCACTGAAAGCCCAGGCAGGCAAATGAGGACCATTGGTCACTCTGGCCAGCTGCCACAGCGAAAAGGGAAAGGCAGTGGGTTGA
 >chr19:42404832-42405112 + E=1e-138 p=0e+00
GGAATCCTCACTGGTTTTTTTTTTTTTGAGACGATCTCGCTCTGTCGCCTAGGCTAGAGTGCAGTGGCGTGATCTCCGCTCACTGCAAC

CTCCACCTCCCAGGTTCAAGCGATTGTCCTGCCTCAGCCTCCCAAGTAGCTGGAACTACAGGTGCCCACCACCACTCCTGGCTAA|TTT
                                                                                      >c
TT|TTGAGACAGAGTCTCATTCTGTTGCCCAGGCTGGAGTGAAGTGGCAAAATCTTGGCTCACTGCAACCTCCACCTCCCAGGTTCAAG
hr19:36325956-36326108 - E=1e-79
CGATTCTTTTGCCTTAGCCTCCCGAGTTGCTGGGATTACAGGCACCTGCCACCACACCTGG|TA|GCATTGAAGGGGGAGGGTGGCTGG
                                                                >chr19:38903917-3890416
GGCGGGGACAGAGGAGCACAGTTAGTCACTGCATAGGCTTAGGGGGGGTTCGAGGTAATTGGGGGAGTCCGAGGCCAGGGGTTGGGTCT
5 + E=2e-137
CACCAGGCTCTTGGGACAACGCGGCTCCCGGGCATAAGAAAGCTCATAGATCTCGTCTTCCGTGTAGAAGAGGTCCAGGGAGAGCTGGA

GCAGACAGGGGTGTTACGGATCCTCCCATCTATAGCCCTGCCCCAG
```
